# The course of peripheral neuropathy and its association with health-related quality of life among colorectal cancer patients

**DOI:** 10.1007/s11764-020-00923-6

**Published:** 2020-11-13

**Authors:** Cynthia S. Bonhof, Lonneke V. van de Poll-Franse, Dareczka K. Wasowicz, Laurens V. Beerepoot, Gerard Vreugdenhil, Floortje Mols

**Affiliations:** 1 Department of Medical and Clinical Psychology, CoRPS - Center of Research on Psychology in Somatic disorders, Tilburg University, Tilburg, The Netherlands; 2 Department of Research, Netherlands Comprehensive Cancer Organisation (IKNL), Utrecht, The Netherlands; 3 Division of Psychosocial Research and Epidemiology, The Netherlands Cancer Institute, Amsterdam, The Netherlands; 4 Department of Surgery, Elisabeth-TweeSteden hospital, Tilburg, the Netherlands; 5 Department of Internal Medicine, Elisabeth-TweeSteden hospital, Tilburg, the Netherlands; 6 Department of Internal Medicine, Máxima Medical Centre, Eindhoven and Veldhoven, The Netherlands

**Keywords:** Peripheral neuropathy, Colorectal cancer, Health-related quality of life, PROFILES

## Abstract

**Purpose:**

To gain more insight into the course of chemotherapy-induced peripheral neuropathy (CIPN) and its impact on health-related quality of life (HRQoL) in a population-based sample of colorectal cancer (CRC) patients up to 2 years after diagnosis.

**Methods:**

All newly diagnosed CRC patients from four hospitals in the Netherlands were eligible for participation in an ongoing prospective cohort study. Patients (*n* = 340) completed questions on CIPN (EORTC QLQ-CIPN20) and HRQoL (EORTC QLQ-C30) before initial treatment (baseline) and 1 and 2 years after diagnosis.

**Results:**

Among chemotherapy-treated patients (*n* = 105), a high sensory peripheral neuropathy (SPN) level was reported by 57% of patients at 1 year, and 47% at 2-year follow-up, whereas a high motor peripheral neuropathy (MPN) level was reported by 47% and 28%, at years 1 and 2, respectively. Linear mixed model analyses showed that SPN and MPN symptoms significantly increased from baseline to 1-year follow-up and did not return to baseline level after 2 years. Patients with a high SPN or MPN level reported a worse global quality of life and a worse physical, role, emotional, cognitive, and social functioning compared with those with a low SPN or MPN level.

**Conclusions:**

Future studies should focus on understanding the mechanisms underlying CIPN so targeted interventions can be developed to reduce the impact of CIPN on patient’s lives.

**Implications for cancer survivors:**

Patients need to be informed of both CIPN and the impact on HRQoL.

Colorectal cancer (CRC) is the third most common cancer among men and women [1]. In 2019, 12,900 patients were diagnosed in the Netherlands. Fortunately, survival rates have improved remarkably as a result of earlier detection and improved treatment strategies. In the past 20 years, the 5-year survival rate has increased from 54 to 66% in the Netherlands [2]. The increasing number of CRC survivors highlights the need to focus on the side effects of cancer and its treatment.

A common and severe side effect is chemotherapy-induced peripheral neuropathy (CIPN), which is the result of damage to the peripheral nerves caused by chemotherapy. The prevalence differs depending on type of chemotherapeutic agent and method of assessment. However, a meta-analysis reported an overall prevalence of 68% in the first month after chemotherapy, and 30% at 6 months or more [3]. There is currently no strategy available to prevent CIPN and pharmacological options to manage established CIPN are limited [4]. Therefore, the development of severe CIPN is often a reason for dose reduction or even discontinuation of the chemotherapeutic agent, compromising the efficacy of treatment and patient survival [5].

CIPN symptoms in CRC are primarily sensory (e.g., tingling, numbness, and pain in the extremities), but can be motoric (e.g., cramps and loss of strength), or autonomic (e.g., dizziness after standing up and blurry vision) as well [5]. These symptoms can cause problems with regular daily activities, which are likely to compromise health-related quality of life (HRQoL). This was indeed the conclusion of a review on CIPN and HRQoL [6]. Among patients with CRC, several studies examined the relationship between CIPN and HRQoL [6–10]. We previously showed in an analyses of 1643 CRC survivors 2–11 years after diagnosis that neuropathy was negatively related with all scales of the EORTC QLQ-C30 questionnaire [7]. However, most studies, including ours have been cross-sectional. In addition, while several studies among CRC patients have examined the course of CIPN over time, most studies did either not include a CIPN measurement before the start of chemotherapy, did not examine the course longer than 1 year after diagnosis, or did not distinguish between sensory (SPN), motor (MPN), and autonomic (APN) peripheral neuropathy (PN) [11–13].

Gaining more insight into the course of CIPN and its influence on HRQoL is important to be able to inform and guide CRC patients and clinicians in their decision-making regarding treatment. Therefore, our aim is to prospectively examine (1) the prevalence and course of SPN, MPN, and APN and (2) their association with HRQoL among a population-based sample of CRC patients from diagnosis up to 2 years after diagnosis.

## Methods

### Setting and participants

The PROCORE study is an ongoing prospective, population-based study among CRC patients, aimed to examine the impact of CRC and its treatment on patient-reported outcomes. Data collection was performed within PROFILES (Patient Reported Outcomes Following Initial Treatment and Long Term Evaluation of Survivorship), a registry for the physical and psychosocial impact on cancer and its treatment [14]. PROFILES is directly linked to the Netherlands Cancer Registry (NCR) that collects data from all newly diagnosed cancer patients [2]. Patients were recruited from four Dutch hospitals: Elisabeth-TweeSteden hospital, Catharina hospital, Elkerliek hospital, and Máxima Medical Centre.

All patients newly diagnosed with CRC as a primary tumor between January 2016 and January 2019 were invited to participate. Exclusion criteria were the following: previous cancer diagnosis (except for basal cell carcinoma), cognitive limitations, and the inability to read or write Dutch. All eligible patients were included shortly after diagnosis, before the start of initial treatment. However, some patients who were previously diagnosed with cancer and those who already started treatment were also included. Therefore, patients were excluded for analysis if (1) they were previously diagnosed with cancer and reported baseline EORTC QLQ-CIPN20 scores > 0, or (2) they already started chemotherapy.

### Data collection

From their research nurse or case manager, patients received an information package, containing an information letter, informed consent form, and the first questionnaire. In the first questionnaire, patients could indicate if they wanted to receive the follow-up questionnaires online. After patients had provided their consent, follow-up questionnaires were sent 4 weeks after surgery (when applicable), and 1 and 2 years after diagnosis. As the questionnaire 4 weeks after surgery did not contain questions on CIPN, it was not included in the analysis. The PROCORE study was approved by the Medical research Ethics Committees United (approval number NL51119.060.14).

### Sociodemographic and clinical characteristics

Patients’ sociodemographic (i.e., age, sex) and clinical information (e.g., cancer type, clinical stage, surgery (yes/no), chemotherapy (yes/no), and radiotherapy (yes/no)) was obtained from patients’ medical records by the NCR [2]. Educational level and partner status were assessed in the questionnaire. Comorbidity was assessed with the adapted Self-administered Comorbidity Questionnaire [15].

### PN

PN symptoms were assessed with the EORTC QLQ-CIPN20 [16], which contains three subscales, assessing SPN, MPN, and APN symptoms. Items are measured on a Likert scale, ranging from (1) not at all to (4) very much. Scores are transformed to a 0–100 scale, with higher scores representing more complaints [17].

Because PN symptoms can also be present in people without cancer, which are then associated with comorbidity and normal aging [18], we wanted to distinguish patients with a high PN level from those with symptoms in the normal range. Therefore, Dutch age and sex-specific normative EORTC QLQ-CIPN20 data [18] and the minimal clinically important difference (MCID) of the CIPN20 (i.e., 2.5 for SPN and 2.6 for MPN) were used [19]. For example, in the general population, men aged 60–69 years reported a mean SPN score of 2.9. Therefore, in the current study, men in this age group were categorized into “high SPN” if they had a sensory score of ≥ 5.4 (2.9 + 2.5), and into “low SPN” if they reported scores < 5.4. No categorization was done for APN, as no MCID is available [19].

### Health-related quality of life

The EORTC QLQ-C30 was used to assess HRQoL [20]. In this study, only the global health status/QoL scale and the five functioning scales were used. Items are scored on a Likert scale from (1) not at all to (4) very much, except for the global QoL scale, which ranges from (1) very poor to (7) excellent. Scores were linearly transformed to a 0–100 scale, with higher scores representing better QoL/functioning [17].

#### Statistical analyses

NCR data on sociodemographic and clinical characteristics enabled us to compare eligible patients and respondents, and those who completed baseline and either 1-year follow-up or 2-year follow-up with patients who completed all questionnaires, using *t* tests for continuous variables and chi-square (or Fisher’s exact) tests for categorical variables. All other analyses are based on patients who completed at least two questionnaires. First, differences in patient characteristics between patients who received chemotherapy and those who did not were assessed with *t* tests for continuous variables and chi-square tests for categorical variables.

Then, logistic regression analyses were performed to detect differences in PN symptoms at 1-year and 2-year follow-up between patients who were given chemotherapy and those who were not. For these analyses, the individual items of the EORTC QLQ-CIPN20 were used and the answer categories “quite a bit” and “very much” were combined. Analyses were adjusted for age, diabetes mellitus, osteoarthritis, and rheumatoid arthritis, which are variables known or expected to impact PN.

The SPN, MPN, and APN courses were examined using linear mixed models (LMM), with maximum likelihood estimation and an unstructured covariance matric with a 2-level structure (i.e., repeated time points [lower level], patients [higher level]). Time was analyzed as a regular categorical predictor with three levels (i.e., three time points). These analyses were adjusted for age, diabetes mellitus, osteoarthritis, and rheumatoid arthritis. Differences in SPN, MPN, and APN between patients with or without chemotherapy were examined similarly, but without time as a predictor. Unstandardized regression coefficients (*E*) are reported. Clinically important differences were determined using the MCID [19].

To detect differences in HRQoL between patients according to the stability of their SPN or MPN levels, *t* tests were conducted. For this, patients were categorized into “ever SPN” if they reported high SPN on at least one of the three time points; otherwise, they were categorized into “never SPN.” The same was done for MPN. Clinically important differences were determined using EORTC QLQ-C30 guidelines [21].

Finally, the impact of SPN and MPN on HRQoL over time was examined with LMM. SPN and MPN were included as dichotomous variables (high vs. low level of SPN/MPN) and analyzed as time-varying predictors, while for the confounding background variables age, sex, partner status, education level, tumor type, stage, osteoarthritis, rheumatoid arthritis, and diabetes mellitus baseline characteristics were used.

Analyses were performed using SPSS 22 (IBM SPSS Statistics for Windows, version 22.0 Armonk, NY: IBM Corps USA). *p* values < 0.05 were considered statistically significant.

## Results

### Patient characteristics

**Fig. 1:**
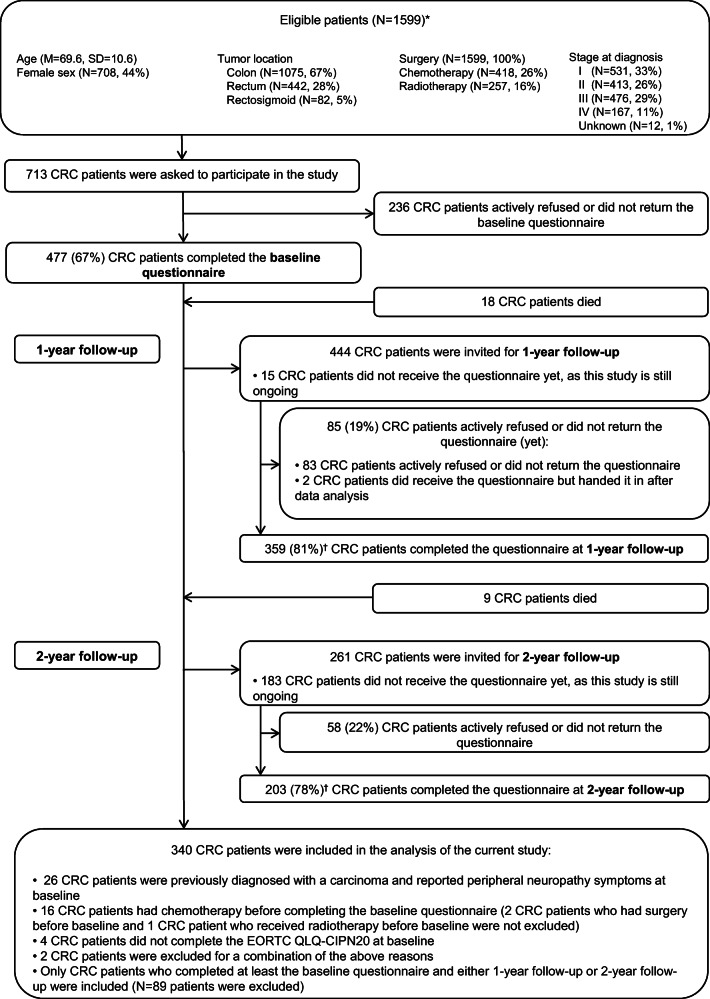
Flowchart of the study. *Characteristics of eligible CRC patients were obtained from the Netherlands Cancer Registry. ^†^Among chemotherapy-treated CRC patients only, 76% completed 1-year follow-up and 79% completed 2-year follow-up

Of the 713 CRC patients who were invited to the study, 66.9% (*n* = 477) completed the questionnaire at baseline, 50.4% (*n* = 359) at 1-year follow-up, and 28.5% (*n* = 203) at 2-year follow-up (Fig. 1.). As the study is still ongoing, not all patients have yet received the questionnaire at 1-year and 2-year follow-up. Compared with all patients eligible for participation, respondents were younger (70 vs. 67 years, *p* < 0.001), more often male (56% vs. 61%, *p* = 0.04), they received chemotherapy more often (26% vs. 31%, *p* = 0.02), and surgery less often (100% vs. 98%, *p* < 0.001). Furthermore, they were less often diagnosed with rectosigmoid cancer (5% vs. 3%, *p* = 0.02), they more often had stage III cancer (29% vs. 37%, *p* = 0.003), and an unknown stage (1% vs. 2%, *p* < 0.001), and less often stage IV cancer (11% vs. 4%, *p* < 0.001). No differences in sociodemographic and clinical characteristics were found between patients who completed at least two questionnaires and patients who completed all three questionnaires (data not shown).

Further analyses were done among respondents who completed at least two questionnaires (*n* = 340). Among these patients, chemotherapy-treated patients (*n* = 105, 31%) were younger, they less often had at least 2 comorbidities, and they more often had a higher disease stage compared with those not treated with chemotherapy (*n* = 235, 69%) (Table 1). They were also more often treated with radiotherapy, but less often received surgery. No data on chemotherapy regimen (e.g., type of chemotherapy administered, number of chemotherapy cycles, and dosage) was available.

**Table 1:** Sociodemographic and clinical characteristics at baseline of colorectal cancer patients, stratified by chemotherapy

	Chemotherapy (*n* = 105 (31%))	No chemotherapy (*n* = 235 (69%))	*p* value
Age (mean, SD)	63.8 (8.8)	67.5 (8.4)	*< 0.001*
Female sex	33 (31%)	99 (42%)	0.06
Partner (yes)	93 (89%)	197 (84%)	0.29
Education level^a^			0.43
Low	6 (6%)	23 (10%)	
Medium	67 (64%)	146 (63%)	
High	32 (31%)	64 (28%)	
Tumor location			0.99
Colon	76 (72%)	168 (72%)	
Rectum/rectumsigmoid	29 (28%)	66 (28%)	
Colon and rectumsigmoid	0 (0%)	1 (0.4%)	
TNM stage			*< 0.001*
I	1 (1%)	103 (44%)	
II	8 (8%)	86 (37%)	
III	92 (88%)	35 (15%)	
IV	4 (4%)	6 (3%)	
Unknown	0 (0%)	5 (2%)	
Tumor differentiation grade			0.21
Well differentiated	1 (1%)	1 (0.4%)	
Moderately differentiated	90 (86%)	181 (77%)	
Poorly differentiated	4 (4%)	16 (7%)	
Unknown	10 (10%)	37 (16%)	
Radiotherapy (yes)	27 (26%)	26 (11%)	*< 0.001*
Surgery (yes)	98 (93%)	235 (100%)	*< 0.001*
Number of comorbidities			*0.048*
None	33 (32%)	62 (27%)	
One	41 (39%)	71 (31%)	
Two or more	30 (29%)	100 (43%)	
Comorbidities associated with PN^b^			
Osteoarthritis	20 (19%)	53 (23%)	0.47
Rheumatoid arthritis	7 (7%)	13 (6%)	0.68
Diabetes mellitus	5 (5%)	26 (11%)	0.06

Variables may deviate from 100% due to rounding off

*SD* standard deviation

Italicized *p* values indicate statistically significance

^a^Education: low (no or primary school); medium (lower general secondary education or vocational training); high (pre-university education, high vocational training, university)

^b^Most frequent comorbidities associated with peripheral neuropathy

### Peripheral neuropathy

At 1-year follow-up, chemotherapy-treated patients reported tingling fingers or hands, tingling toes or feet, numbness in fingers or hands, numbness in toes or feet, and trouble handling small objects significantly more often compared with those not treated with chemotherapy (Table 2). At 2-year follow-up, they still more often reported tingling toes or feet and numbness in toes or feet.

**Table 2:** Peripheral neuropathy symptoms among colorectal cancer patients at 1-year and 2-year follow-up, stratified by chemotherapy

	Year 1			Year 2		
	CT (*n* = 99)	No CT (*n* = 226)	*p* value	CT (*n* = 59)	No CT (*n* = 131)	*p* value
Sensory symptoms and problems						
1. Tingling fingers or hands	25 (25%)	13 (6%)	*< 0.001*	7 (12%)	7 (5%)	0.28
2. Tingling toes or feet	36 (36%)	10 (4%)	*< 0.001*	10 (17%)	5 (4%)	*0.008*
3. Numbness in fingers or hands	15 (15%)	2 (1%)	*< 0.001*	3 (5%)	2 (2%)	0.28
4. Numbness in toes or feet	20 (20%)	5 (2%)	*< 0.001*	7 (12%)	4 (3%)	*0.02*
5. Shooting or burning pain in fingers or hands	6 (6%)	4 (2%)	0.16	2 (3%)	2 (2%)	0.31
6. Shooting or burning pain in toes or feet	8 (8%)	7 (3%)	0.07	5 (9%)	6 (5%)	0.20
9. Trouble standing or walking	10 (10%)	4 (2%)	*0.002*	4 (7%)	4 (3%)	0.99
10. Trouble distinguishing temperature of hot and cold water	2 (2%)	2 (1%)	0.53	2 (3%)	0 (0%)	0.99
18. Trouble hearing	9 (9%)	20 (9%)	0.50	6 (10%)	11 (8%)	0.42
Motor symptoms and problems						
7. Cramps in hands	4 (4%)	7 (3%)	0.79	2 (3%)	7 (5%)	0.67
8. Cramps in feet	4 (4%)	7 (3%)	0.76	4 (7%)	6 (5%)	0.47
11. Trouble holding a pen which made writing difficult	1 (1%)	4 (2%)	0.50	1 (2%)	2 (2%)	0.93
12. Trouble handling small objects (e.g., buttoning a blouse)	15 (15%)	9 (4%)	*< 0.001*	5 (9%)	6 (5%)	0.10
13. Trouble opening jar/bottle due to loss of strength in hands	12 (12%)	17 (8%)	0.28	3 (5%)	11 (9%)	0.59
14. Trouble walking because your feet come down to hard	3 (3%)	1 (0.4%)	0.08	2 (3%)	1 (1%)	0.27
15. Trouble walking stairs or standing up from a chair due to weakness in legs	8 (8%)	11 (5%)	0.28	3 (5%)	4 (3%)	0.54
19. Only for those driving cars: Trouble driving due to use of pedals	2 (2%)	2 (1%)	0.60	2 (4%)	0 (0%)	0.99
Autonomic symptoms and problems						
16. Dizziness after standing up	5 (5%)	9 (4%)	0.60	1 (2%)	5 (4%)	0.53
17. Blurry vision	2 (2%)	8 (4%)	0.83	1 (2%)	4 (3%)	0.69
20. Only for men: Trouble getting or maintaining an erection	20 (30%)	40 (35%)	0.79	12 (32%)	33 (47%)	0.21

Italicized *p* values indicate statistically significance. The number of patients reported in this table reflects the patients who answered “quite a bit” or “very much” on the EORTC QLQ-CIPN20 items

Analyses were adjusted for age, diabetes mellitus, osteoarthritis, and rheumatoid arthritis

The prevalence of a high SPN and MPN level (using MCID + normscores) was also examined. While for SPN, no differences between chemotherapy-treated patients and those without chemotherapy were found at baseline (11% vs. 16%; *p* = 0.17); chemotherapy-treated patients did more often report a high SPN level at 1-year (57% vs. 24%; *p* < 0.001) and 2-year follow-up (47% vs. 31%; *p* = 0.03). Regarding MPN, chemotherapy-treated patients reported a high MPN level more often compared with those not treated with chemotherapy, but only at 1-year follow-up (47% vs. 32%; *p* = 0.02). No differences were found at baseline (15% vs. 23%; *p* = 0.13) and 2-year follow-up (28% vs. 35%; *p* = 0.34).

### Course of peripheral neuropathy

**Fig. 2:**
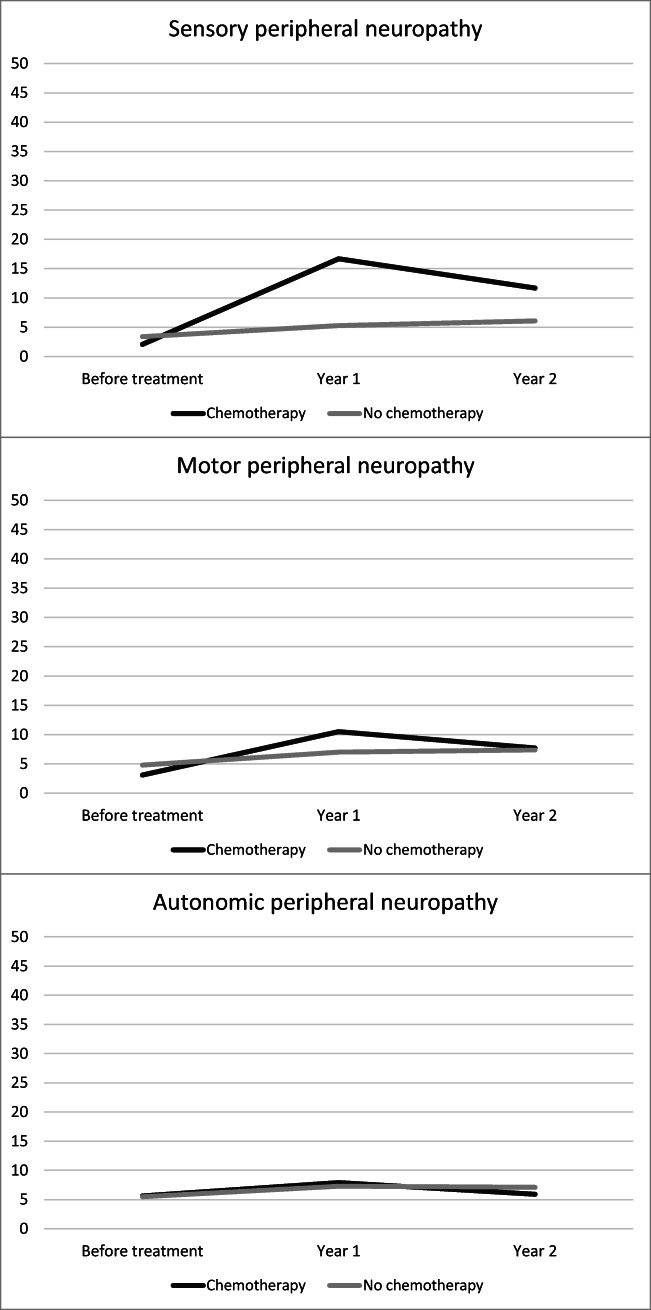
Course of sensory, motor, and autonomic peripheral neuropathy among colorectal cancer patients, stratified by chemotherapy. A higher score on the scales indicates more neuropathy symptoms. The scale in this figure ranges from 0 to 50 for clear visibility of the course of the peripheral neuropathy symptoms, while total scores of the EORTC QLQ-CIPN20 range from 0 to 100

For chemotherapy-treated patients, SPN increased significantly at 1-year follow-up. Furthermore, while the SPN mean score then did significantly decrease at 2-year follow-up, it remained significantly higher compared with baseline. All changes in mean score were significant (all three *p* < 0.001) and clinically relevant (Fig. 2). For patients not treated with chemotherapy, SPN increased at 1-year (*p* = 0.001, not clinically relevant) and at 2-year follow-up remained stable, but still significantly higher compared with baseline (*p* < 0.001, clinically relevant). Finally, chemotherapy-treated patients reported a higher SPN mean score at 1-year (*p* < 0.001) and 2-year follow-up (*p* = 0.001). These differences were clinically relevant.

For MPN, the mean score increased for both chemotherapy-treated patients and those not treated with chemotherapy at 1-year (both *p* < 0.001), and at 2-year follow-up remained stable and still significantly higher compared with baseline (*p* = 0.004 and *p* < 0.001). However, the significant differences were only clinically relevant for chemotherapy-treated patients. Moreover, chemotherapy-treated patients reported a higher MPN mean score, but only at 1-year follow-up (*p* = 0.007, clinically relevant).

Finally, APN showed a stable course among chemotherapy-treated patients, while for those not treated with chemotherapy, there was a small increase at 1-year follow-up (*p* = 0.048, not clinically relevant). No differences were found between chemotherapy-treated patients and those not treated with chemotherapy.

### Peripheral neuropathy and HRQoL

**Fig. 3:**
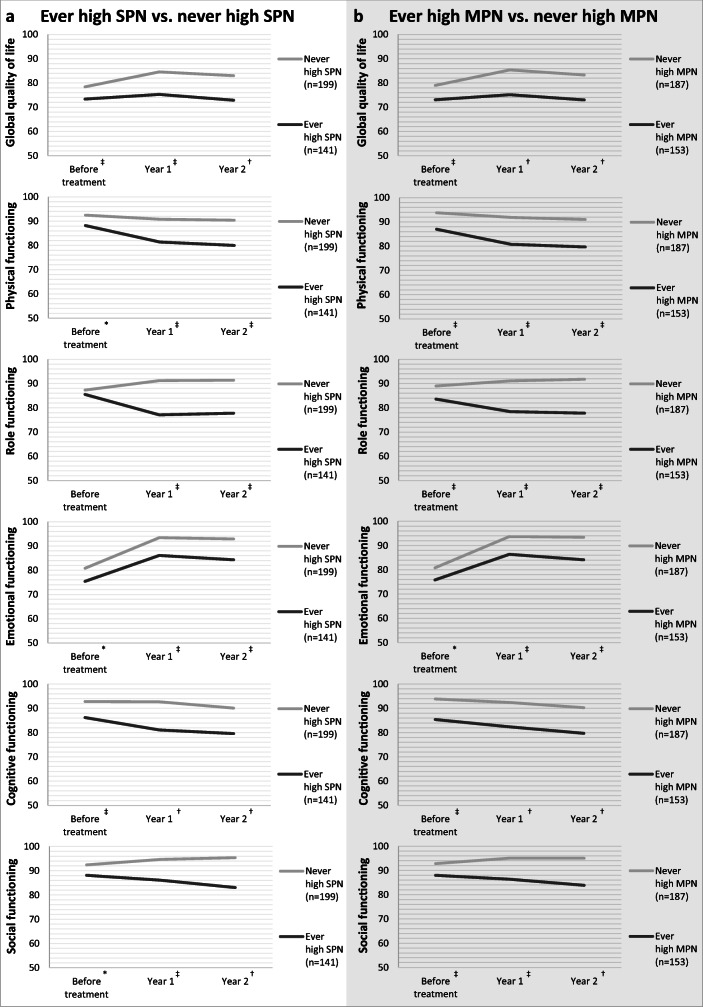
Course of health-related quality of life for colorectal cancer patients according to them ever or never reporting high sensory peripheral neuropathy (**a**) or motor peripheral neuropathy (**b**). *Significant difference between patients who “ever” reported high SPN/MPN and those who “never” reported high SPN/MPN, but of no clinical relevance. ^‡^Significant difference, of small clinical relevance. ^†^Significant difference, of medium clinical relevance. ^¥^Significant difference, of large clinical relevance. The scale in this figure ranges from 50 to 100 for clear visibility of the association between peripheral neuropathy and health-related quality of life, while total scores of the EORTC QLQ-C30 range from 0 to 100

In further analyses, both patients who received chemotherapy and those who did not were included in the analyses. At baseline, patients in the “ever high SPN” group reported worse global quality of life, and worse physical, emotional, cognitive, and social functioning compared with patients who never reported high SPN (Fig. 3). However, only the difference in global quality of life and cognitive functioning were of (small) clinical relevance. At 1-year and 2-year follow-up, patients in the “ever high SPN” group reported worse scores on all six HRQoL scales, which were of small to medium clinical relevance.

For MPN, patients in the “ever high MPN” reported worse scores on all six HRQoL scales at all three time points (Fig. 3). However, at baseline, only the differences in global quality of life and physical, cognitive, and social functioning were of (small) clinical relevance. At 1- and 2-year follow-up, all differences were clinically relevant (small to medium).

### Between-patients and within-patients effects of peripheral neuropathy on HRQoL

CRC patients with a high SPN level reported a worse global quality of life (*E* = − 11.87, *p* < 0.001) and a worse physical (*E* = − 11.34, *p* < 0.001), role (*E* = − 11.46, *p* < 0.001), emotional (*E* = − 9.59, *p* < 0.001), cognitive (*E* = − 13.43, *p* < 0.001), and social functioning (*E* = − 10.62, *p* < 0.001) compared with those with a low SPN level (between-patients effects). Furthermore, patients who changed from a low to a high SPN level over time showed a decrease in physical (*E* = − 8.24, *p* < 0.001), role (*E* = − 8.83, *p* < 0.001), cognitive (*E* = − 6.47, *p* < 0.001), and social (*E* = − 5.72, *p* = 0.001) functioning, while patients who changed from a high to a low SPN level over time showed improvements in these areas (within-patients effects).

For MPN, those with a high MPN level also reported a worse global quality of life (*E* = − 11.86, *p* < 0.001) and a worse physical (*E* = − 14.16, *p* < 0.001), role (*E* = − 13.35, *p* < 0.001), emotional (*E* = − 8.36, *p* < 0.001), cognitive (*E* = − 12.81, *p* < 0.001), and social (*E* = − 11.10, *p* < 0.001) functioning compared with those with a low MPN level. In addition, patients who changed from a low to a high MPN level over time showed a decrease in global quality of life (*E* = − 6.62, *p* < 0.001) and physical (*E* = − 10.59, *p* < 0.001), role (*E* = − 14.91, *p* < 0.001), cognitive (*E* = − 5.41, *p* < 0.001), and social (*E* = − 7.59, *p* < 0.001) functioning, while those who changed from a high to a low MPN level over time showed improvements on these scales.

## Discussion

In this longitudinal study among CRC patients, we first showed that, at 1-year follow-up, chemotherapy-treated patients more often reported tingling fingers or hands, tingling toes or feet, numbness in fingers or hands, numbness in toes or feet, and trouble handling small objects, compared with those not treated with chemotherapy. At 2-year follow-up, they still more often reported tingling and numbness in toes or feet. These results are in line with prior research, in which it was also found that symptoms in the hands are more prominent during and shortly after chemotherapy, while symptoms in the feet are more prominent months after chemotherapy [7, 22, 23]. Also, the overall CIPN prevalence is reported to be 30% at 6 months or more after chemotherapy [3]. While in this study the prevalence rate of MPN at 2-year follow-up (28%) supports those findings, the reported SPN (47%) is much higher.

Looking at the course of PN, both SPN and MPN were impacted by chemotherapy, and the reported SPN and MPN did not return to baseline level after 2 years. However, at 2-year follow-up, the MPN level did decline to the same level as those not treated with chemotherapy. The finding that SPN was mostly impacted by chemotherapy is supported by previous studies [7, 11, 13]. The question remains whether SPN symptoms will continue to decrease after 2 years, or that it remains a chronic problem after this period of time. The small increase in PN symptoms in those not treated with chemotherapy could be due to older age and an increase in age- and PN-related comorbidity, such as diabetes mellitus, rheumatoid arthritis, and osteoarthritis. Also, we only had data on chemotherapy as primary treatment while it is possible that patients in the “no chemotherapy” group did receive chemotherapy as secondary treatment.

Regarding the association between PN and HRQoL, CRC patients with a high SPN or MPN level reported a worse global quality of life and a worse functioning compared with those with a low level. Previous cross-sectional studies among CRC patients have found similar results. For example, our previous study among CRC survivors 2–11 years after diagnosis showed that those with many neuropathy symptoms reported significant and clinically relevant worse HRQoL scores on all EORTC QLQ-C30 subscales [7]. Another study among oxaliplatin-treated CRC survivors up to 7 years post chemotherapy also found that PN was associated with worse HRQoL [8].

The results of this study regarding both the long-lasting course of SPN and the impact of PN on HRQoL indicate that it is crucial to inform patients and clinicians about CIPN and its impact on patients’ lives. In addition, currently no preventive treatment for CIPN is available. More studies aiming to improve our understanding of the mechanisms underlying the development of CIPN are needed, so targeted interventions can be developed. Several alternative chemotherapy options are available to prevent (severe) CIPN. Evidence shows that shortened durations (3 vs. 6 months) of FOLFOX or CAPOX chemotherapy can be given to stage III colon cancer patients, without compromising survival [24]. CAPOX is preferred, as it results in a lower incidence of long-lasting SPN compared with FOLFOX chemotherapy [25]. For established (painful) CIPN, duloxetine is the only agent recommended in the treatment of CIPN [4]. In addition, preliminary empirical evidence suggests that non-pharmacological treatments such as exercise [26] and cognitive-behavioral therapy [27] may be effective in preventing and/or treating (painful) CIPN. More studies are needed to provide more evidence for the effectiveness of these non-pharmacological treatments. Also, future research should examine possible sociodemographic, clinical, and psychological factors that predict the onset and persistence of CIPN.

The present study has some limitations. First, data on type of chemotherapy, number of chemotherapy cycles, and dose reduction were not available, while these factors are important determinants of CIPN and thereby could have impacted our results [3]. The lack of a clinician-based assessment of CIPN is another limitation, as patient-reported assessments of CIPN should preferably be combined with clinician-rated neurological assessment tools [28]. However, especially patient-reported assessment seems important, as healthcare professionals often underestimate the severity and frequency of neuropathy symptoms [29]. Furthermore, generalization of the results of this study should be done with caution, as eligible patients and the respondents of this study did show some differences in sociodemographic and clinical characteristics. Moreover, as the PROCORE study is still ongoing, not all patients received the questionnaires at 1-year and 2-year follow-up. While no differences in sociodemographic and clinical characteristics were found between patients who completed both baseline and either 1-year or 2-year follow-up and those who completed all three questionnaires, this could have impacted (the strength of) our findings. Finally, it remains unknown whether those lost to follow-up stopped participating due to PN in their hands. If so, this may have resulted in an underestimation of our findings.

Despite these limitations, this is, to the best of our knowledge, the first longitudinal study that examined the association between CIPN and HRQoL among CRC patients up to 2 years after diagnosis. In addition, by examining not only between-patients effects but also within-patient effects in the association between CIPN and HRQoL, a stronger support for causality is provided.

In conclusion, the results of this study indicate that especially SPN symptoms are still prevalent 2 years after diagnosis. In addition, both SPN and MPN were significantly associated with a worse HRQoL. Therefore, it is crucial that patients are informed of both CIPN and the impact on HRQoL. Due to the currently limited treatment options, clinicians may also offer support to CRC survivors by monitoring for the symptoms and supporting survivors in their search for a solution, for example, by informing them about possible benefits of exercise and cognitive-behavioral therapy. Future studies should focus on increasing the understanding of the mechanisms underlying both the development of CIPN and CIPN chronicity, so targeted interventions can be developed to reduce the impact of CIPN on patients’ lives.

## Data Availability

The data that support the findings of this study are available from the profiles registry (www.profilesregistry.nl).

## Abbreviations

PROFILES: Patient Reported Outcomes Following Initial treatment and Long term Evaluation of Survivorship
PROs: Patient-reported outcomes
NCR: Netherlands Cancer Registry
HRQoL: Health-related quality of life
CIPN: Chemotherapy-induced peripheral neuropathy
PN: Peripheral neuropathy
SPN: Sensory peripheral neuropathy
MPN: Motor peripheral neuropathy
MPN: Autonomic peripheral neuropathy
CRC: Colorectal cancer
